# A new device (FAQ.FIX®) for orthodontic bracket placement in straight wire technique

**DOI:** 10.1186/2196-1042-14-23

**Published:** 2013-08-19

**Authors:** Francesco Mazzeo, Edoardo Marchese, Valeria Assumma, Joseph Sepe, Letizia Perillo

**Affiliations:** Dipartimento Multidisciplinare Di Specialita’ Medico-Chirurgiche Ed Odontoiatriche, School of Specialty of Orthodontics, Second University of Naples, Via Luigi De Crecchio 6, Naples, 80138 Italy; Private Practice, via Roma, 158, Salerno, 84121 Italy; Private Practice, via G. Bruno, 69, Naples, 80122 Italy; Biological Sciences, University of Maryland, College Park, MD 20742 USA

## Abstract

**Background:**

In straight wire preadjusted appliances, all the information required to position the teeth in three planes is included in the brackets placed at the midpoint of the facial axis of the clinical crown, defined by facial axis point (FA). Central to this technique is the bracket placement.

Preadjusted orthodontic appliances cannot get the right tooth position with a straight wire because of the inaccuracy of bracket placement. Horizontal, axis, vertical, and base are the most common bracket placement errors.

The aim of this paper was to describe a bracket positioner to fix the brackets accurately (Q) on FA point (FAQ.FIX®) in direct or indirect bonding.

**Methods:**

After the development of a prototype, a FAQ.FIX® along with a Bracket Placement Clinical Chart was developed and thus described.

**Results:**

FAQ.FIX® along with the Bracket Placement Clinical Chart may facilitate the accuracy in bracket placement on FA point avoiding the most common bracket placement errors regardless the operator skill, even in particular or difficult case.

**Conclusions:**

FAQ.FIX®may represent a significant improvement in the bracket placement compared to the bracket eye and the traditional gauges positioning. Further studies will be needed to verify the clinical efficacy.

## Background

The straight wire technique is the appliance used most widely in orthodontic therapy today. It was introduced by Andrews [1] in 1972. The basic premise of the preadjusted system is that proper bracket position allows the teeth to be placed with a straight wire into an occlusal contact with an excellent mesiodistal inclination (tip) and excellent faciolingual inclination (torque) [2].

All the information required to position a tooth in three planes is included in the brackets placed at the midpoint of the facial axis of the clinical crown (FACC), defined by facial axis point (FA). Over the last 40 years, several changes have been made to Andrews' appliance [3, 4] with improvements in preadjusted appliances, without bends on the archwire, to achieve the ideal alignment and leveling, but the most important phase is still the bracket placement [1].

Poorly positioned brackets result in poorly placed teeth and necessitate more archwire adjustments. This can lead to an increased treatment time or poor occlusion [5]. Several studies [2, 6–9] have demonstrated that the preadjusted orthodontic appliances cannot get the right tooth position with a straight wire because of the poor bracket placement.

Unfortunately, even under the best of circumstances, the ideal bracket placement during initial bonding is often impossible because of the existing malocclusion, operator error, or tooth structure variation [4, 5, 9–12]. Horizontal, axis, vertical, and base are the most common bracket placement errors [2, 4, 6–9]. The ‘eyeball’ bracket position, described by Andrews, cannot be considered reliable and satisfying, as well as, the positioning with the help of a gauge [2, 6–9]. Because of bracket placement errors, orthodontists still spend considerable time in detailing to get the proper alignment of crowns and roots and leveling marginal ridges, particularly near the end of the treatment, to compensate them. Currently, no placement method, direct or indirect, can guarantee the correct execution of this delicate procedure [6, 7, 13–15].

The aim of this paper was to describe a bracket positioner to fix the brackets accurately (Q) on FA point (FAQ.FIX®) in direct or indirect bonding.

## Methods

### FAQ.FIX® prototype

In straight wire appliance, the ideal positioner should have the following:

- An adjustable locking mechanism for height
- References to identify the FACC
- An insert for the slot which reproduce the bracket angulation

The torque, instead, included in the base of the bracket, is expressed by base inclination.

**Figure 1:**
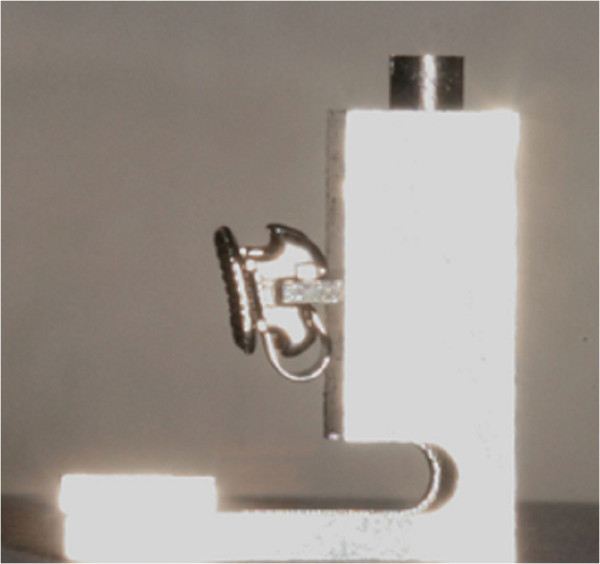
**L-shaped FAQ.FIX® prototype with horizontal and vertical arms and insert for the slot reproducing bracket angulation.**

**Figure 2:**
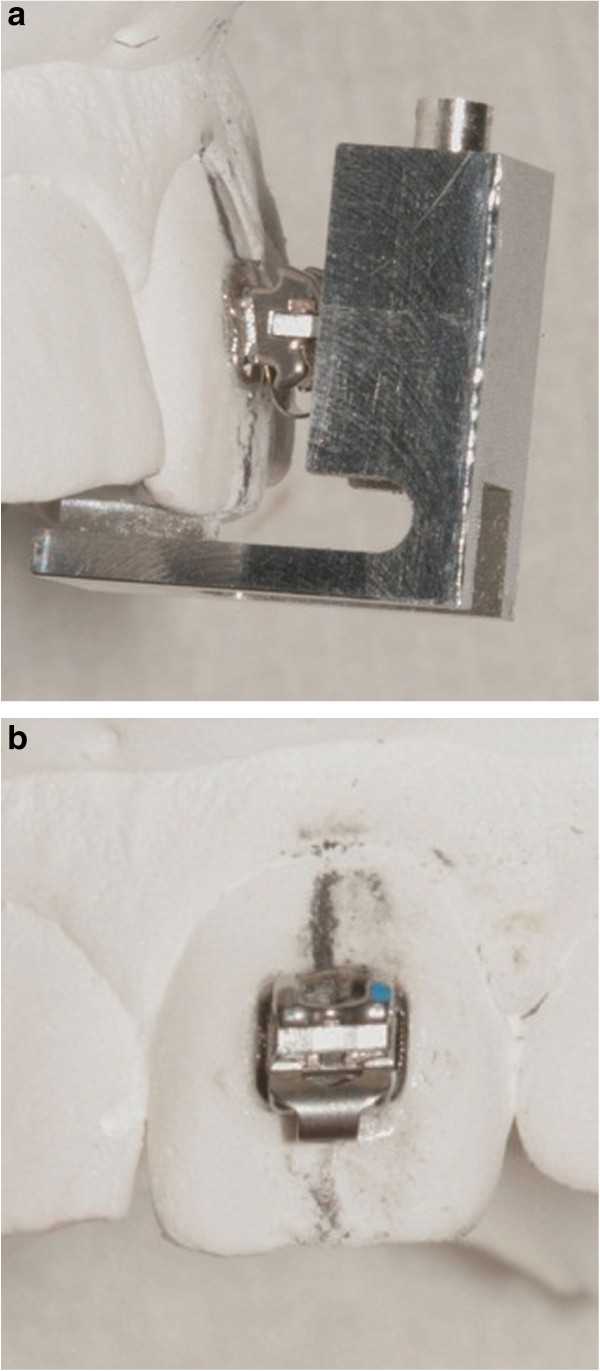
**Placements of the FAQ.FIX® prototype.** **(a)** Lateral view of the placement. **(b)** Bracket placed with FAQ.FIX® prototype on an upper left central incisor of a stone model.

The first prototype of the FAQ.FIX® (Cnc Lab Tech, Salerno, Italy) positioner was L-shaped due to horizontal and vertical arms. The insert for the slot reproducing the bracket angulation for each tooth was located on the back and was thick enough to hold the bracket (Figure 1). Developed to place a 5° preangled bracket on an upper left central incisor of a stone model, with a 10-mm FACC, the FAQ.FIX® prototype, without an adjustable locking mechanism for height, had the insert for the slot fixed at 5 mm from the occlusal edge with the same bracket 5° angulation. The FAQ.FIX® prototype worked as tweezers to place the bracket on the selected tooth with the mark on the upper border of the bracket base, and the one on the center of the base of the prototype fell on the FACC (Figure 2). A light-cured composite adhesive was applied to the base.

The FAQ.FIX® prototype test confirmed the accuracy of the bracket placement.

### FAQ.FIX® features

**Figure 3:**
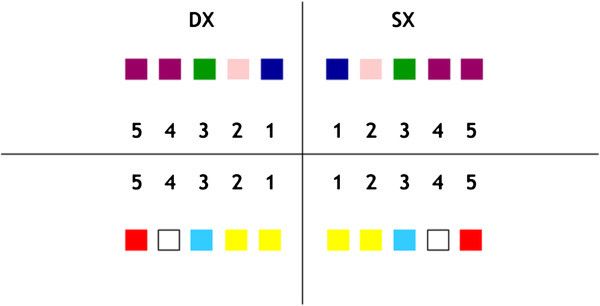
**International color coding.** Upper central incisors, dark blue; upper lateral incisors, pink; upper cuspids, green; upper bicuspids, violet; lower incisors, yellow; lower cuspids, light blue; lower first bicuspids, white; lower second bicuspids, red.

**Figure 4:**
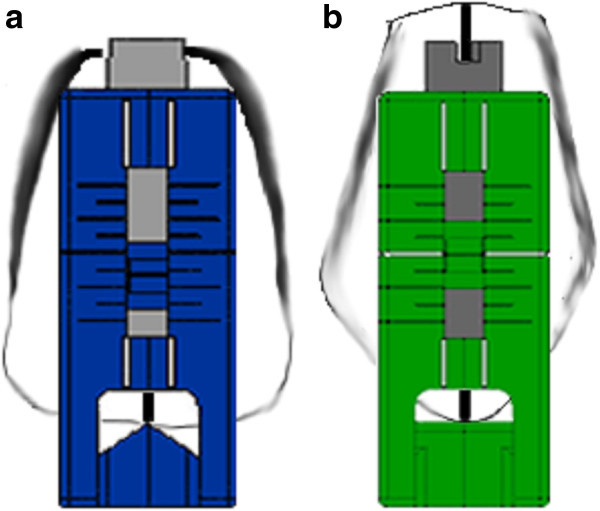
**Two types of FAQ. FIX®. (a)** For flat edge teeth as incisors and **(b)** for cuspidate teeth.

After FAQ.FIX® prototype test, the kit of 20 positioners was produced, one for each tooth and reproducing the preinserted angulation of the single prescription, identified by the international color coding and a distogingival dot (Figure 3). To improve the accuracy and get a unique contact point between the base of the positioner and the tooth, two types of positioners were made, the former for flat edge teeth as the incisors (Figure 4a) and the latter for cuspidate teeth (Figure 4b). Horizontal reference lines were drawn on the vertical arm to set the desired height, whereas vertical reference lines were drawn on the horizontal and vertical arms to identify the FACC.

**Figure 5:**
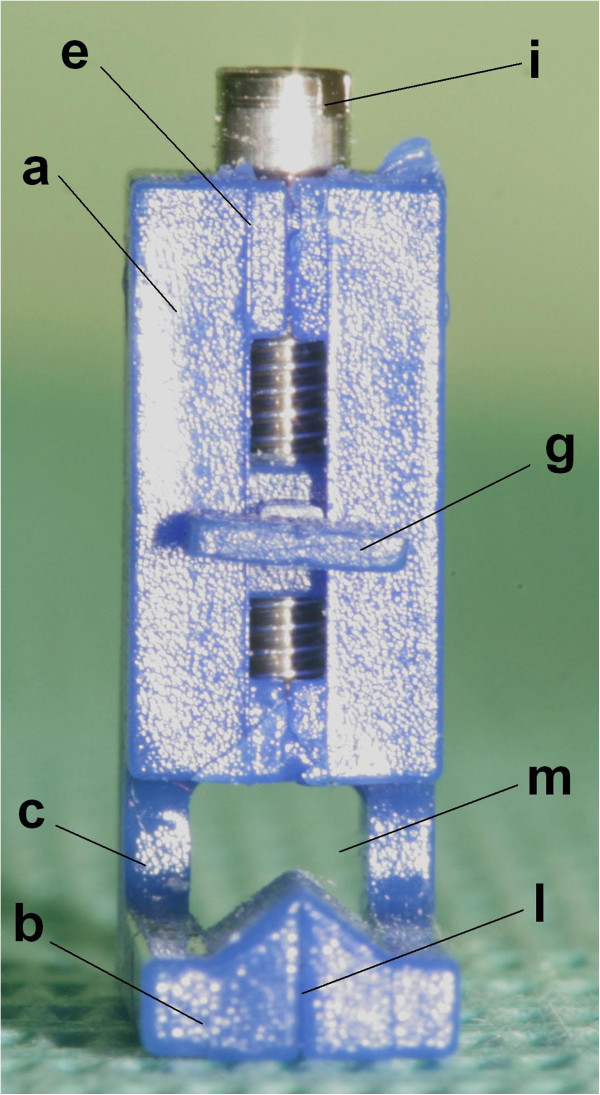
**FAQ.FIX® posterior surface view. (a)** Vertical arm. **(b)** Horizontal arm. **(c)** Connection arms between vertical and horizontal arms. **(e)** Vertical reference lines on the vertical arm. **(g)** Insert for the slot. **(i)** Screw. **(l)** Vertical reference lines on the horizontal arm. **(m)** Hole to better place the bracket on the FACC of the tooth.

**Figure 6:**
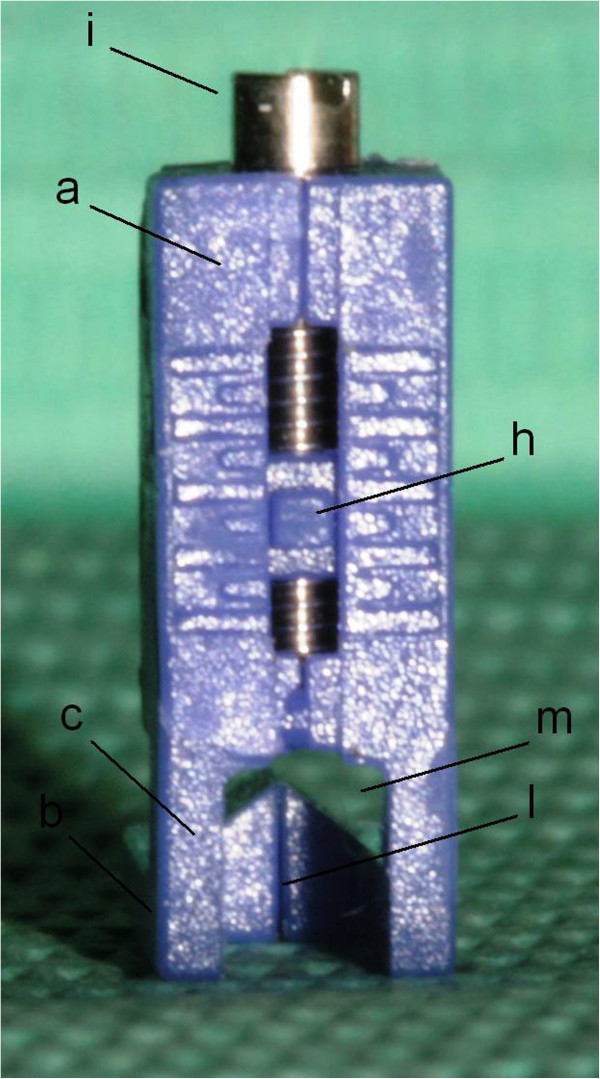
**FAQ.FIX® anterior surface view. (a)** Vertical arm. **(b)** Horizontal arm. **(c)** Connection arms between vertical and horizontal arms. **(h)** Insert for the slot. **(i)** Screw. **(l)** Vertical reference lines on the horizontal arm. **(m)** Hole to better place the bracket on the FACC of the tooth.

**Figure 7:**
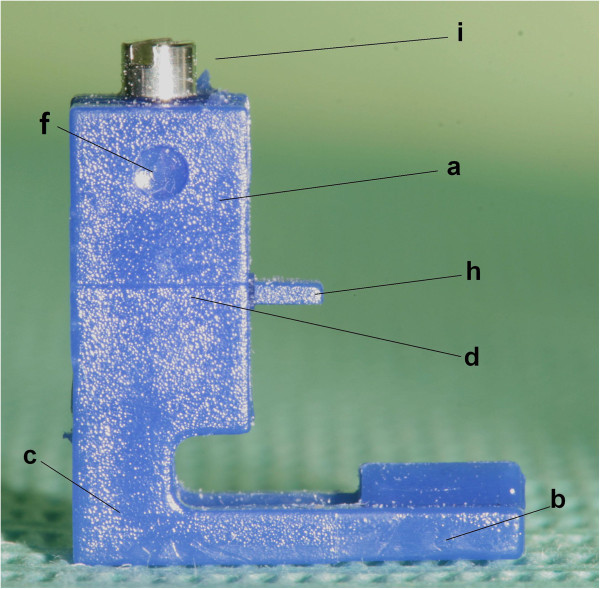
**FAQ.FIX® lateral surface view. (a)** Vertical arm. **(b)** Horizontal arm. **(c)** Connection arms between vertical and horizontal arms. **(d)** Horizontal reference line on the vertical arm. **(f)** Distogingival dot. **(h)** Insert for the slot. **(i)** Screw.

A screw was positioned on the upper surface to make the mechanism for height adjustable from 3 to 7 mm from the occlusal edge, with 0.5 mm variations (Figures 5, 6 and 7).

The FAQ.FIX® is designed exclusively for straight wire brackets with 0.022 × 0.028 in slot; it is made of polycarbonate for medical use (Bayer Material Science AG, Leverkusen, Germany), and the screw is made of best quality steel. Positioners and screw are made of atoxic material, cold sterilizable and autoclavable at 121° with the ‘plastic instruments’ or ‘delicate’ program for thermosensitive materials.

**Figure 8:**
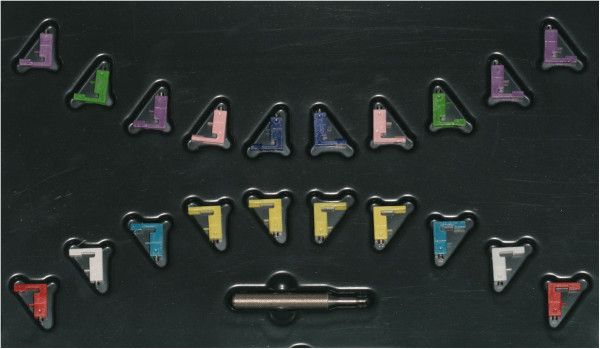
**The FAQ.FIX® kit with 20 positioners and the screwdriver supplied.**

Each FAQ.FIX® kit (Figure 8) is individually designed for the single prescription and producer. Using FAQ.FIX®, the orthodontist has to follow the instructions carefully, placing it on the tooth, without watching the bracket, and align it to the FACC drawn on the tooth.

In an ideal bracket placement, the FAQ.FIX® positioner allows the match between the center of the insert and the FA point.

### Bracket placement clinical chart

**Figure 9:**
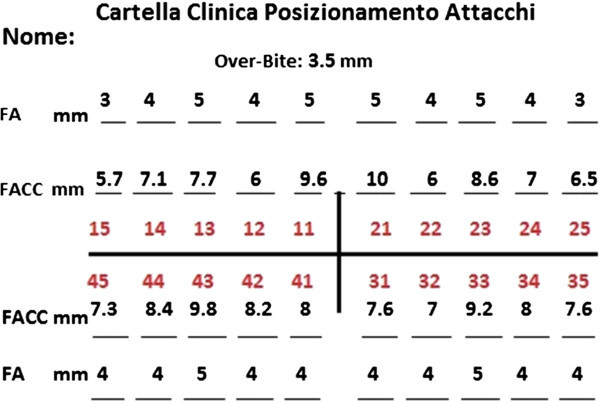
**Bracket placement clinical chart.**

**Figure 10:**
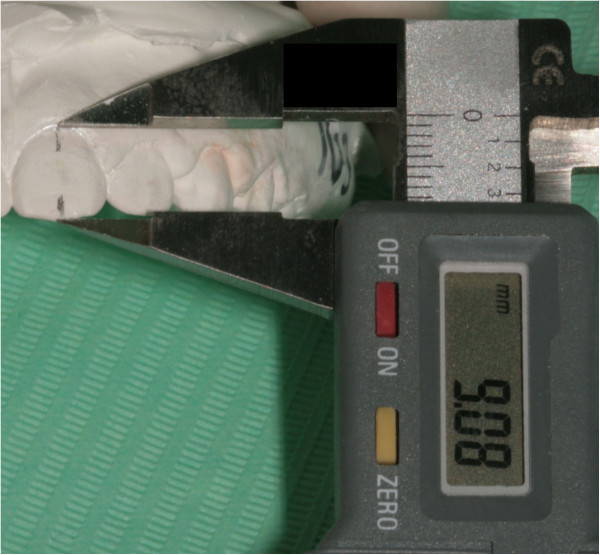
**Measurement of the FACC of an upper central incisor using an electronic gauge.**

The use of a positioning tool in straight wire techniques requires measuring the height of the crown of the teeth, so a ‘Bracket Placement Clinical Chart,’ to keep in the patient's record (Figure 9), has to be developed using the following guidelines:

– Measure the height of the crown of each tooth (Figure 10) and report it on the corresponding FACC row

If the same teeth (i.e., cuspids) do not have the same height, due to incomplete eruption or coronal fracture, it may be useful to refer to the completely erupted and/or intact tooth [4, 16–18] (i.e., incisors).

- Report the selected FA points for each tooth on FA row
- Report the patient overbite to eventually adjust the placement as always done [4].

For the development of the Bracket Placement Clinical Chart, the operator can also use the MBT or the AACD criteria [4, 16–18], measuring only the height of an upper central incisor to obtain the correct height of the other teeth.

### Operative sequence of FAQ.FIX® direct or indirect placement bracket (Figure 11)

**Figure 11:**
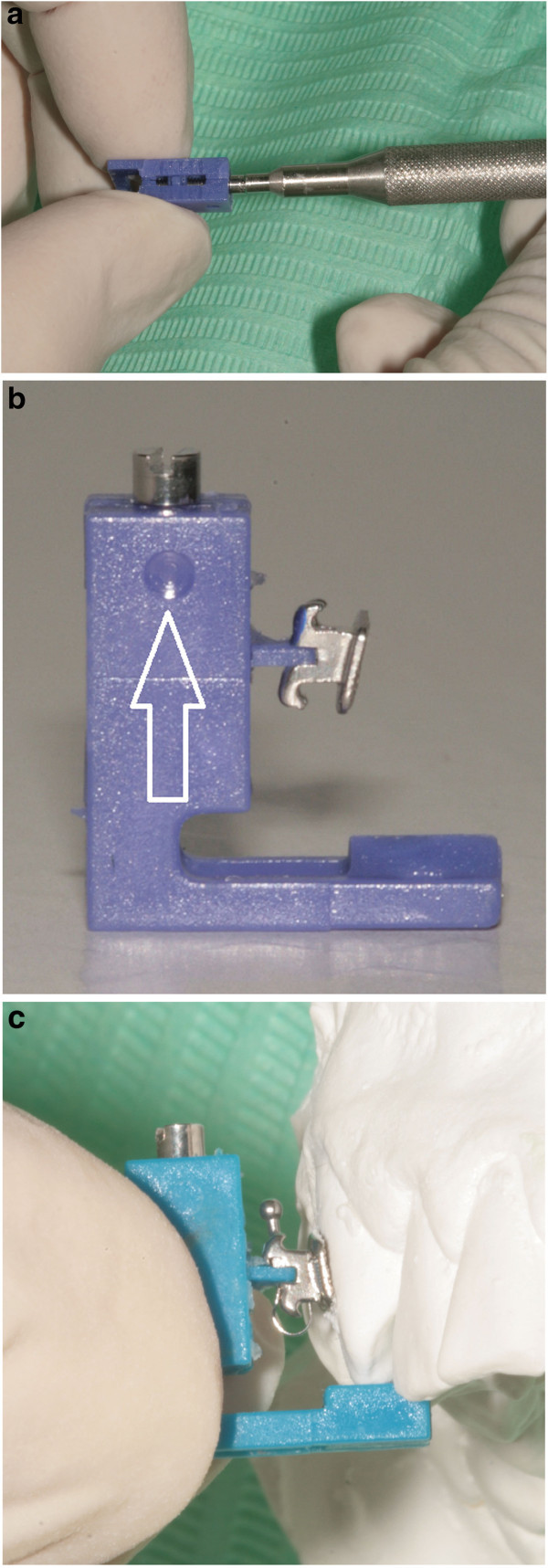
**Operative sequence of FAQ.FIX® bracket placement. (a)** Adjustment of the height with a screwdriver. **(b)** Introduction of the insert into the bracket's slot. **(c)** Placement of the bracket on the tooth.

Using the Bracket Placement Clinical Chart:

- Adjust the height with the screwdriver supplied (Figure 11a).
- Introduce the insert into the bracket's slot (Figure 11b).
- Apply a light-cured adhesive on the bracket base.
- Apply the bracket on the tooth using FAQ.FIX® as tweezers (Figure 11c).
- Remove the excess composite.
- Polymerize
- Remove FAQ.FIX®.

### Indirect bonding bracket placement

**Figure 12:**
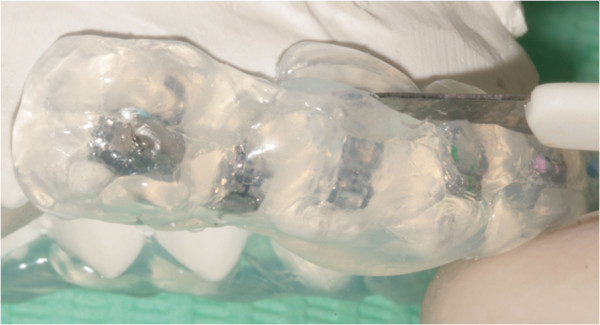
**The transfer tray made for the indirect bonding bracket placement.**

**Figure 13:**
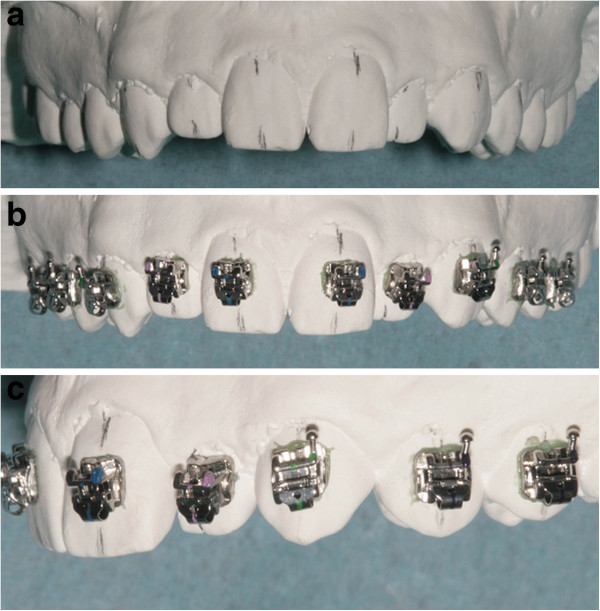
**Indirect bonding bracket placement with upper lateral incisors and cuspids partially erupted. (a)** Frontal view of the stone model with the FACC drawn. **(b** and **c)** Frontal and lateral views to highlight the accuracy of the indirect bonding, respectively.

The brackets were positioned using FAQ.FIX® on the stone model and then applied accurately on each patient's tooth using a simple and efficient system of indirect bonding that can be easily performed in the orthodontic office using a transfer tray (Figure 12) [19]. An example of indirect bonding bracket placement is reported in Figure 13.

### Direct bonding bracket placement

**Figure 14:**
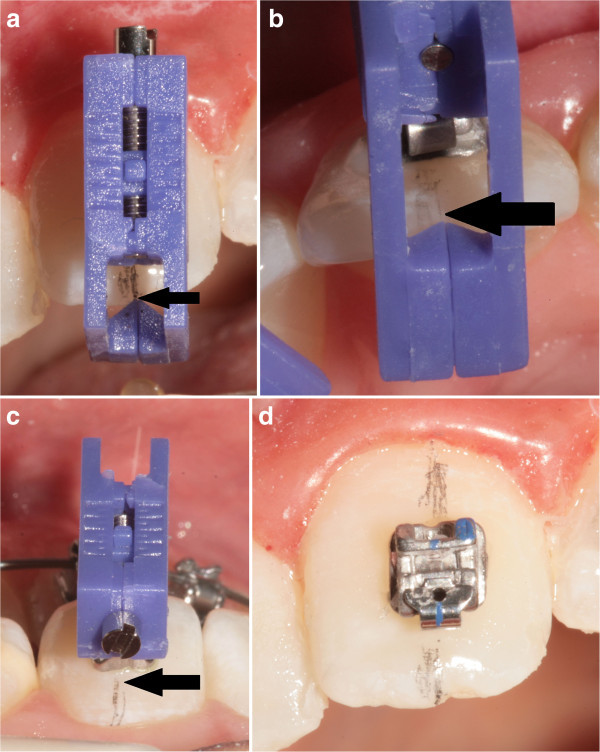
**Direct bonding bracket placement. (a, b, and c) Frontal, occlusal, and gingival views of the direct placement, respectively. (d)** Bracket accurately placed on the FA point.

**Figure 15:**
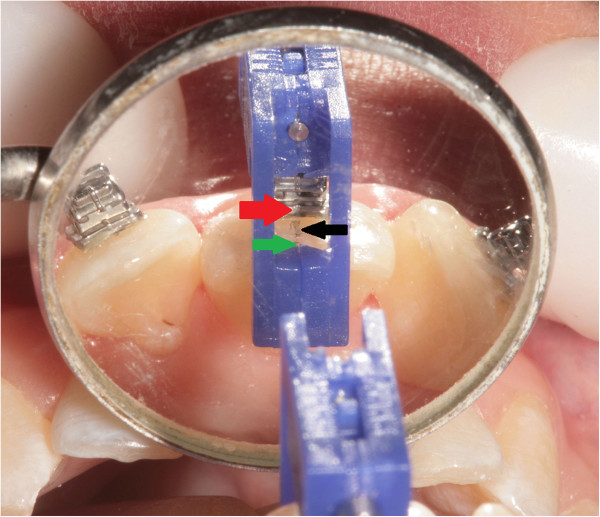
**Direct bonding bracket placement.** View from the mirror black arrow, FACC; red arrow, reference on the bracket's base; green arrow, center of FAQ.FIX®.

The brackets were positioned using FAQ.FIX® on the teeth, using the same reference used above. The view of the tooth and of FACC line drawn on the tooth, through the ‘window,’ that acts as a viewfinder is easy (Figures 14 and 15).

### Operating time

- FACC measurement time of 10 teeth: almost 3 min.
- FAQ.FIX® positioners adjustment with 10 brackets insertion: almost 5 min.
- Direct/indirect bonding bracket placement: depends by the operator's skill, but according to the authors, it could be faster than the eyeball bracket position or using a gauge.

Written informed consent was obtained from the patient for the publication of this report and any accompanying images.

## Results and discussion

The aim of any orthodontist is to place the teeth in an ideal position. In straight wire technique, this should be achieved with preadjusted orthodontic appliance [1]. Unfortunately, an orthodontist may fail this goal even with a preadjusted orthodontic appliance [2, 6–9, 13].

Factors such as bracket placement errors, tooth structure variation [2, 6–10], archwire bending adjustments, and/or rebonding bracket positions are frequently required, and thus, bracket placement remains central to straight wire technique.

The FAQ.FIX® was developed to facilitate the accuracy in bracket placement on FA point.

The FAQ.FIX® along with the Bracket Placement Clinical Chart allows orthodontists to avoid the most common bracket placement errors regardless of operator skill, even in case of partially or fractured teeth or in more difficult cases, such as Class II, Division 2 [11, 12, 20].

The FAQ.FIX®, tested more than 3 years ago and used by the authors and other orthodontists in hundreds of bondings, confirmed the accuracy and validity of systematics.

The authors recommend FAQ.FIX® in the indirect bonding because the bracket placement on a stone model using this positioner is easier and more accurate. The transfer, using transfer trays, on patient's teeth will be fast (almost 15 min including wire insert for self-ligating brackets) and accurate [19].

Direct bonding bracket placement using FAQ.FIX® may increase the chair time, but it addresses errors of initial bracket positioning decreasing treatment times and obtaining superior results.

The FAQ.FIX® is easy to use, safe, inexpensive, and quick to learn. Moreover, it is a useful teaching tool for young orthodontists, focusing their attention on the bracket placement accuracy.

## Conclusions

Correct bracket placement is a problem for both expert and beginning orthodontists. The failure may compromise the treatment outcome. The FAQ.FIX® may represent a significant improvement in bracket placement compared to the eyeball and the traditional gauges bracket position. The authors have verified treatment time reduction using this device because of the initial placement error decrease.
